# Clinical Characteristics and Prognosis of Patients With Pulmonary Mucoepidermoid Carcinoma: A SEER-Based Analysis

**DOI:** 10.3389/fonc.2021.601185

**Published:** 2021-03-05

**Authors:** Lingxiao Qiu, Pan Song, Pingmei Chen, Huaqi Wang, Fangfang Li, Mengxuan Shu, Gen-cheng Gong, Xiangjin Song, Chun Huang, Hongxia Jia, Nana Li, Guojun Zhang

**Affiliations:** ^1^ Department of Respiratory Medicine, The First Affiliated Hospital of Zhengzhou University, Zhengzhou, China; ^2^ Academy of Medical Sciences, Zhengzhou University, Zhengzhou, China; ^3^ Department of Urology, West China Hospital of Sichuan University, Chengdu, China; ^4^ Department of Neonatology, West China Guang’an Hospital, Sichuan University, Guang’an, China; ^5^ Zhengzhou Key Laboratory for Chronic Respiratory Disease, Zhengzhou, China; ^6^ Henan Provincial Respiratory Medicine Center, Zhengzhou, China; ^7^ The First Clinical Medical College of Lanzhou University, Lanzhou, China; ^8^ State Key Laboratory of Respiratory Disease, National Clinical Research Center for Respiratory Disease, Guangzhou Institute of Respiratory Health, The First Affiliated Hospital of Guangzhou Medical University, Guangzhou, China

**Keywords:** pulmonary mucinous epidermoid carcinoma, clinical characteristics, prognosis, Surveillance, Epidemiology, and End Results (SEER), nomogram

## Abstract

**Background:**

Primary pulmonary mucoepidermoid carcinoma (PMEC) is an extremely rare malignancy. Its clinical characteristics and prognosis are not fully understood. This study evaluated clinical characteristics and prognostic factors of PMEC and established a nomogram to predict its 1-, 3-, 5- and 10-year cancer-specific survival (CSS) rates.

**Methods:**

In the Surveillance, Epidemiology, and End Results database from January 1, 1975 to December 31, 2016, patients pathologically diagnosed with PMEC were identified. Kaplan–Meier analysis and Cox regression were performed to evaluate the CSS stratified by different covariates. A predictive nomogram model was built and validated by the concordance index (C-index) and calibration curves.

**Results:**

A total of 585 PMEC patients were identified. A total of 408 (70%) of patients were placed into the training cohort, and 177 (30%) patients were placed into the validation cohort. The 5- and 10-year CSS rates of stage I–II PMEC patients were 91.4 and 88.9, respectively. The 1-, 3- and 5-year CSS rates of stage III–IV PMEC were 56.5, 39.45, and 32.1%, respectively. Survival curves showed that older age, large tumor size, poor differentiation, and high TNM stage were associated with a significantly worse prognosis. CSS outcomes were significantly better in patients who received surgical treatments (surgical alone, surgery plus radiation and/or chemotherapy). Patients who received radiation and/or chemotherapy had the worst prognosis. Multivariate Cox results revealed that covariates, including age, tumor laterality, tumor sizes, pathological differentiation, lymph node metastasis, distant metastasis, TNM stage and therapy, were independent prognostic factors for PMEC. These factors were used to construct a nomogram. The C-index of the nomogram was 0.921. The calibration curve presented favorable consistency between the predicted CSS and actual observations. This nomogram was validated by the validation cohort. The C-index of the validation cohort was 0.968.

**Conclusion:**

Age, bilateral tumors, tumor size, pathological differentiation grade, lymph node metastasis, distant metastasis, TNM stage and therapy were independent prognostic factors of PMEC patients. The first nomogram for predicting the CSS of PMEC was built and validated, showing its potential value in practice.

## Background

Lung cancer is the most commonly diagnosed cancer in both males and females globally (1–3). It has become the leading cause of cancer-associated death among males in both developed and developing countries, as well as the leading cause of cancer death among females in developed countries (1–3). Mucoepidermoid carcinoma (MEC) is a subtype of salivary gland tumors (SGTs), which represent a group of fairly rare lung tumors (4). It is estimated that MEC accounts for only less than 1% of primary malignant lung neoplasms (5, 6). MEC often occurs in the parotid gland, submandibular gland, sublingual gland and lacrimal gland, and is infrequently seen in the lungs (7–9). Due to its low frequency of pulmonary origination, little is known about the demographics, treatment, survival, or prognostic factors associated with primary pulmonary mucoepidermoid carcinoma (PMEC). To date, the current characterization and prognosis of PMEC have been informed mostly by case reports and small case series (6, 10–12). Even the largest SGT series to date—with 699 MEC patients from the National Cancer Database (NCDB)—did not analyze PMEC patients separately from MEC alone (13). Thus, there is an opportunity to profile the understanding of PMEC by assessing it in a more targeted series.

The Surveillance, Epidemiology, and End Results (SEER) database consists of 18 cancer registries across the United States, which covers approximately 30% of the US population with cancer diagnosis, treatment, and survival data. The extensive tumor patient information in the SEER database has a large advantage for analyzing the features and prognosis of rare cancers. In our study, we aimed to examine the characteristics, survival, and prognostic factors of PMEC patients with the SEER database.

## Materials and Methods

### Data Source

The data in this study were derived from the SEER database with the software SEER*Stat version 8.3.5. Patients diagnosed with primary PMEC were retrospectively identified from January 1, 1975 to December 31, 2016.

### Selection Criteria

Patients were eligible if they met the following criteria: (1) patients were diagnosed with lung cancer; and (2) the diagnosis of PMEC was confirmed by histology or exfoliative cytology.

The following criteria were used for data exclusion: (1) multiple primary tumors; (2) unclear or incomplete follow-up time; and (3) the survival state at the end of follow-up was unclear.

### Variables and Main Outcomes

The following basic characteristics were collected and sorted: age at diagnosis (≤35, 36–60, 61–74, and ≥75), sex (male or, female), race (white, black, and other races, including American Indian and Asian/Pacific Islander), primary tumor site (main bronchus, upper lobe, middle lobe, lower lobe, overlapping lesion of the lung, and lung NOS), tumor laterality (left, right, one side but side unspecified, and bilateral), tumour sizes, pathological differentiation grade (well differentiated, moderate differentiation, poor differentiation, and undifferentiated), T stage (T1, T2, T3, T4, and Tx), N stage (N0, N1, N2, N3, and Nx), M stage (M0, M1, and Mx), therapy (surgery, radiation or chemotherapy, and combined therapy, including surgery + radiation, surgery + chemotherapy, and surgery + radiation +chemotherapy), survival months and survival status (alive, dead from PMEC, and dead from other reasons). Cancer-specific survival (CSS) was the main outcome.

### Statistical Analyses

The included patients were randomly divided into a training cohort (70%) and validation cohort (30%) by the random number methods. Baseline characteristics including age, sex, race, primary tumor site, laterality, tumour sizes, pathological differentiation grade, TNM stage, T stage, N stage, M stage, and therapy, were described. Kaplan–Meier analysis was utilized for the CSS of PMEC patients according to the above factors. The 1-, 5-, 10-, and 20-year CSS rates of PMEC patients with AJCC TNM stages I–II and III–I III–IV were calculated with survival tables. Survival curves were constructed, and hazard ratios (HRs) with 95% confidence intervals (95% CIs) were calculated.

Univariate Cox regression analysis was used to evaluate each variable’s value for predicting CSS. A multivariate Cox regression model was used to analyze variables with P < 0.05 in univariate analyses. A nomogram model was built with the coefficients of each factor in multivariate Cox analysis. The concordance index (C-index) and calibration curves were performed to evaluate the predictive accuracy of the nomogram. Validation of the nomogram model was conducted with the validation cohort.

All statistical analyses were performed with SPSS version 25, R software version 3.2.3 and GraphPad Prism 7.0. P < 0.05 was considered statistically significant.

## Results

### Patient Characteristics

The sociodemographics, tumor attributes, and treatment of PMEC are outlined in **Table 1**. A total of 585 eligible PMEC patients with a median age of 54 (35–68) years were identified. PMEC was more common in younger populations (59.4% of patients were aged less than 60 years *versus* 40.5% of patients were aged more than 60 years). Surgical resection was performed in 83.7% of PMEC tumor patients. Of the patients who were treated without surgery, the majority were treated with chemotherapy and/or radiation (9.4% of all patients). A total of 408 (70%) patients were randomly placed into a training cohort, and 177 (30%) patients were placed into a validation cohort. The median follow-up time of the included patients was 37 (8–124) months.

**Table 1:** Patients’ characteristics of included patients.

Variables	Total (n = 585)	Training cohort (n = 408)	Validation cohort (n = 177)
**Age, n(%)**			
≤35	146(25)	98(24)	48(27.1)
36–60	202(34.5)	144(35.3)	58(32.8)
61–74	16728.5)	118(28.9)	49(27.7)
≥75	70(12.0)	48(11.8)	22(12.4)
**Gender, n(%)**			
Male	309(52.8)	222(54.4)	87(49.2)
Female	276(47.2)	186(45.6)	90(50.8)
**Race, n(%)**			
White	455(77.8)	322(78.9)	133(75.1)
Black	65(11.1)	44(10.8)	21(11.9)
Others	63(10.8)	40(9.8)	23(13)
Unknown	2(0.3)	2(0.5)	0(0)
**Primary Site, n(%)**			
Main bronchus	76(13)	56(13.7)	20(11.3)
Upper lobe	206(35.2)	136(33.3)	70(39.5)
Middle lobe	51(8.7)	34(8.3)	17(9.6)
Lower lobe	182(31.1)	126(30.9)	56(31.6)
Overlapping lesion of lung	24(4.1)	18(4.4)	6(3.4)
Lung, NOS	46(7.9)	38(9.3)	8(4.5)
**Laterality, n(%)**			
Left	269(46)	187(45.8)	82(46.3)
Right	302(51.6)	211(51.7)	91(51.4)
One side but side unspecified	4(0.7)	3(0.7)	1(0.6)
Bilateral	10(1.7)	7(1.7)	3(1.7)
**Tumor sizes, n(%)**			
≤20 mm	118(20.2)	79(19.4)	39(22)
21-30 mm	74(12.6)	49(12)	25(14.1)
31-50 mm	57(9.7)	42(10.3)	15(8.5)
>50 mm	28(4.8)	23(5.6)	5(2.8)
Unclear	308(52.6)	215(52.7)	93(52.5)
**Pathological differentiation, n(%)**			
Well	127(21.7)	79(19.4)	48(27.1)
Moderate	192(32.8)	144(35.3)	48(27.1)
Poor	63(10.8)	44(10.8)	19(10.7)
Undifferentiated	52(8.9)	38(9.3)	14(7.9)
Unclear	151(25.8)	103(25.2)	48(27.1)
**TNM stage, n(%)**			
I	277(47.4)	190(46.6)	87(49.2)
II	29(5)	18(4.4)	11(6.2)
III	50(8.5)	35(8.6)	15(8.5)
IV	70(12)	52(12.7)	18(10.2)
Unclear	159(27.2)	113(27.7)	46(26)
**T stage, n(%)**			
T1	179(30.6)	119(29.2)	60(33.9)
T2	167(28.5)	118(28.9)	49(27.7)
T3	21(3.6)	18(4.4)	3(1.7)
T4	47(8)	35(8.6)	12(6.8)
Tx	78(13.3)	56(13.7)	22(12.4)
Unclear	93(15.9)	62(15.2)	31(17.5)
**N, n(%)**			
N0	342(58.5)	238(58.3)	104(58.8)
N1	35(6)	21(5.1)	14(7.9)
N2	53(9.1)	40(9.8)	13(7.3)
N3	15(2.6)	11(2.7)	4(2.3)
Nx	40(6.8)	31(7.6)	9(5.1)
Unclear	100(17.1)	67(16.4)	33(18.6)
**M, n(%)**			
M0	412(70.4)	288(70.6)	124(70.1)
M1	70(12)	52(12.7)	18(10.2)
Mx	10(1.7)	6(1.5)	4(2.3)
Unclear	93(15.9)	62(15.2)	31(17.5)
**Therapy, n(%)**			
Surgery	388(66.3)	271(66.4)	117(66.1)
Radiation/chemotherapy	55(9.4)	42(10.3)	13(7.3)
Combined therapy	102(17.4)	68(16.7)	34(19.2)
No defined therapy	40(6.8)	27(6.6)	13(7.3)

Combined therapy: surgery plus radiation and/or chemotherapy. NOS: Not otherwise specified.

### Survival Outcomes

#### OS and CSS Rates

The OS and CSS rates of PMEC patients varied greatly with TNM stage (**Table 2**). For the patients with stage I–II PMEC, the 3-, 5- and 10-year OS rates were 88.3, 85.5, and 79.6, respectively. The 3-, 5-, and 10- year CSS rates were 94.7, 94.2, and 91.4%, respectively. For the patients with stage III–IV PMEC, the 1-, 3- and 5- year OS rates were 47.7, 28.2, and 23.8%, respectively. The 1-, 3- and 5- year of CSS rates were 56.5, 39.45, and 32.1%, respectively.

**Table 2:** 1-, 3-, 5- and 10-year overall survival and cancer-specific survival rates of patients with PMEC stages I–II and stages III–IV.

Survival rates		Overall survival	Cancer-specific survival
**Stages I**–**II**	1 year	94.9% (92.35–97.45%)	97.5% (95.74–99.26%)
	3 year	88.3% (84.38–92.22%)	94.7% (91.96–97.44%)
	5 year	85.5% (81.19–89.81%)	94.2% (91.26–97.14%)
	10 year	79.6% (74.31–84.89%)	91.4% (87.48–95.32%)
**Stage III**–**IV**	1 year	47.7% (38.49v56.91%)	56.5% (47.09–65.91%)
	3 year	29.3% (20.68v37.92%)	37.9% (27.9–47.9%)
	5 year	23.8% (15.57–32.03%)	32.1% (22.1–42.1%)
	10 year	0%	25.7% (14.33–37.07%)

#### CSS Curves Stratified by Different Factors

**Figure 1:**
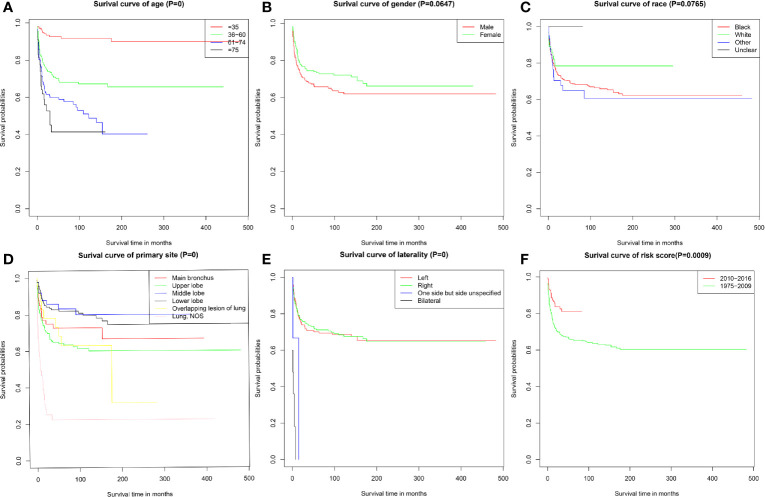
Cancer-specific survival of PMEC patients stratified by **(A)** age; **(B)** gender; **(C)** race; **(D)** primary site; **(E)** laterality; **(F)** time.

As shown in **Figure 1A**, the prognosis for PMEC patients decreased significantly with age (P < 0.001). There was no association between prognosis and sex (**Figure 1B**) or race (**Figure 1C**). PMEC patients with tumors originating in the upper lobe had a worse prognosis than those with primary tumors in the middle or lower lobes (**Figure 1D**). The CSS of PMEC patients with bilateral tumors was significantly shorter than that of PMEC patients with unilateral tumors (P < 0.001) (**Figure 1E**). Differences were observed in the survival curve between the periods of 2010–2016 and 1975–2009 (**Figure 1F**).

**Figure 2:**
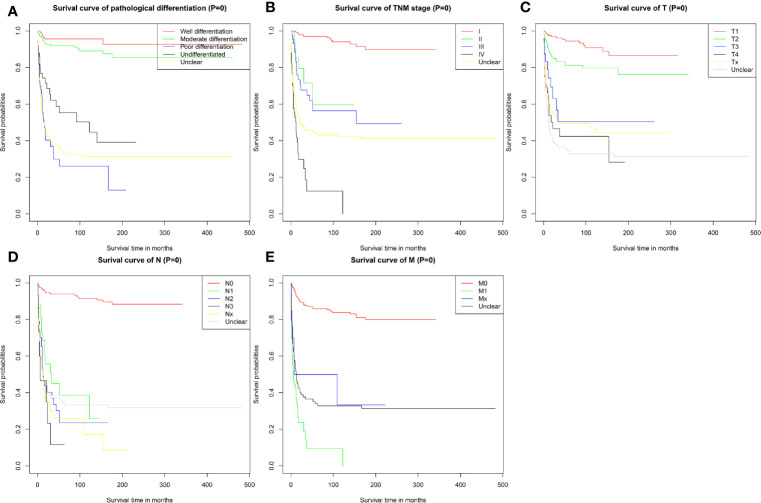
CSS for patients with PMEC stratified by **(A)** pathological differentiation grade; **(B)** TNM stage; **(C)** T stage; **(D)** N stage; **(E)** M stage.

The survival curve also revealed that PMEC patients with poorly differentiated or undifferentiated tumors had much worse survival outcomes than those with highly or moderately differentiated tumors (P < 0.001) (**Figure 2A**). Patients with more advanced TNM stages had obviously worse survival outcomes (**Figure 2B**). With TNM stage I as the reference, the HRs and 95%CIs of stages II, III, and IV were 6.68 (2.72–16.41), 9.70 (5.0–18.83) and 34.33 (18.99–62.06), respectively. Patients with stage T3 and T4 disease had significantly worse survival outcomes than those with stage T1 and T2 disease (P < 0.001) (**Figure 2C**). The prognosis for PMEC patients was much worse for patients with the invasion of lymph nodes (P < 0.001) (**Figure 2D**). The CSS of PMEC patients with distant metastases was significantly shorter than that of PMEC patients without distant metastases (P < 0.001) (**Figure 2E**).

#### Survival Outcomes of Treatments

**Figure 3:**
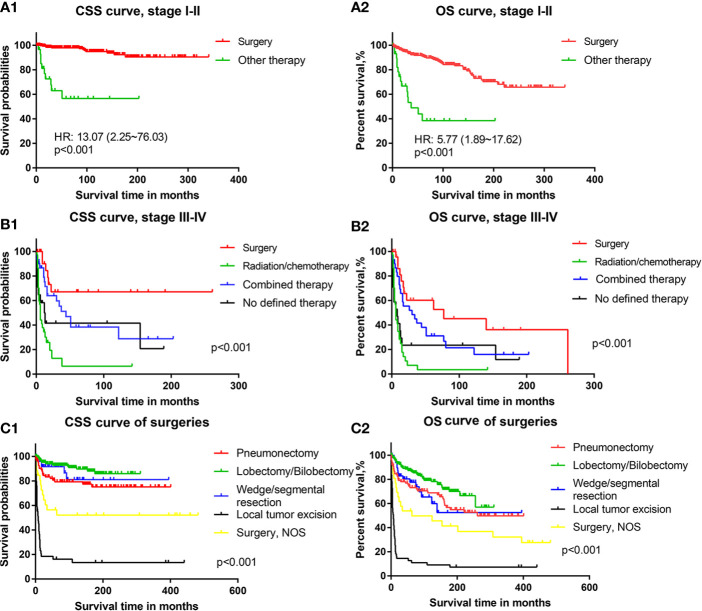
The cancer-specific survival and overall survival of patients with different treatments: **(A1)** Cancer-specific survival of PMEC patients in TNM stages I–II with surgery or other therapy. **(A2)** Overall survival of PMEC patients in TNM stages I–II with surgery or other therapy. **(B1)** Cancer-specific survival of PMEC patients in TNM stages III–IV with surgery, radiation/chemotherapy, combined therapy and no defined therapy. **(B2)** Overall survival of PMEC patients in TNM stages III–IV with surgery, radiation/chemotherapy, combined therapy and no defined therapy. **(C1)** Cancer-specific survival of PMEC patients with pneumonectomy, lobectomy/bilobectomy, wedge/segmental resection and local tumor excision. **(C2)** Overall survival of PMEC patients with pneumonectomy, lobectomy/bilobectomy, wedge/segmental resection and local tumor excision.

For PMEC patients with TNM stage I–II, the results of survival curves showed that patients who underwent cancer-directed surgery had obviously better survival outcomes than those who underwent other treatments (radiation and/or chemotherapy, surgery plus radiation and/or chemotherapy) [CSS HR: 13.07, 95% CI (2.25–76.03), **Figure 3A1**; OS HR: 5.77, 95% CI (1.89–17.62), **Figure 3A2**].

For stage III–IV PMEC patients, the survival results showed that surgical therapy was associated with the highest CSS rate, followed by combined surgery plus radiation and/or chemotherapy. Radiation and/or chemotherapy alone was the worst among all selective therapies (**Figures 3B1, B2**). With surgical therapy as the reference, the CSS HRs (95% CIs)of combined therapy and radiation/chemotherapy were 2.29 (1.01–5.20) and 5.79 (2.95–11.39), respectively. For the OS of PMEC patients with stage III–IV, there was no significant difference between surgery and combined therapy. Survival outcomes of radiation and/or chemotherapy were still the worst among all therapies. With surgical therapy as the reference, the HR and 95% CI of combined therapy and radiation and/or chemotherapy were 1.74 (0.90–3.37) and 3.95 (2.16–7.22), respectively.

Among patients undergoing surgical treatments, those who received pneumonectomy, lobectomy/bilobectomy and wedge/segmental resection had an excellent long-term CSS (**Figure 3C1**). Patients receiving lobectomy/bilobectomy were associated with better prognoses than those receiving pneumonectomy. Prognoses of patients who underwent local tumor excision were the worst among all methods of surgery. With lobectomy/bilobectomy therapy as the reference, the CSS HR and 95% CI of pneumonectomy, wedge/segmental resection and local tumor excision were 2.37 (1.20–4.67), 1.61 (0.58–4.62) and 17.87 (8.13–39.31), respectively. Similar results were revealed by the OS curve of patients with PMEC (**Figure 3C2**). Patients with local tumor excision had a poor prognosis in terms of both CSS outcomes (**Figure 3C1**) and OS outcomes (**Figure 3C2**). With lobectomy/bilobectomy therapy as the reference, the OS HR and 95% CI of pneumonectomy, wedge/segmental resection and local tumor excision were 1.68 (1.06–2.65), 1.80 (0.90–3.61) and 9.92 (5.13–19.18), respectively.

### Univariate and Multivariate Cox

Univariate and multivariate Cox results revealing potential prognostic factors were presented in **Table 3**. Multivariate Cox analysis revealed that elderly age (>60 years), bilateral tumors, large tumor sizes (>30 mm), poorly differentiated and undifferentiated tumors, lymph node metastases and distant metastases were independent prognostic factors of worse survival in PMEC patients (**Table 3**). Conversely, cancer-directed surgery was an independent protective prognostic factor for PMEC patients (**Table 3**).

**Table 3:** Multivariate Cox regression analysis of prognostic factors for patients with pulmonary mucoepidermoid carcinoma.

Characteristic	Univariate			Multivariate		
	HR	95%CI	P value	HR	95%CI	P value
**Age**						
≤35	1		Ref.	1		Ref.
36–60	4.34	(2.33–8.06)	<0.001	1.73	(0.9–3.35)	0.103
61–74	7.34	(3.97–13.57)	<0.001	3.56	(1.85–6.83)	<0.001
≥75	9.5	(4.8–18.81)	<0.001	4.14	(1.92–8.9)	<0.001
**Gender**						
Male	1		Ref.			
Female	0.75	(0.56–1.02)	0.068			
**Race**						
white	1		Ref.			
Black	0.66	(0.37–1.16)	0.148			
others	1.11	(0.69–1.8)	0.664			
Unclear						
**Number of tumors**						
1	1		Ref.	1		Ref.
≥2	0.26	(0.14–0.47)	<0.001	0.16	(0.08–0.3)	<0.001
**Primary Site**						
Main bronchus	1		Ref.			
Upper lobe	1.33	(0.8–2.21)	0.277			
Middle lobe	0.56	(0.25–1.23)	0.148			
Lower lobe	0.63	(0.36–1.11)	0.11			
Overlapping lesion of lung	1.29	(0.59–2.86)	0.524			
Lung, NOS	4.28	(2.41–7.58)	0.277			
**Laterality**						
Left	1		Ref.			
Right	0.98	(0.72–1.34)	0.905	0.95	(0.68–1.33)	0.765
Bilateral	12.62	(5.93–26.84)	<0.001	2.84	(1.24–6.51)	0.014
**Tumor sizes**						
≤20 mm	1		Ref.	1		Ref.
21–30 mm	0.61	(0.22–1.72)	0.354	1.36	(0.46–4.07)	0.581
31–50 mm	2.47	(1.14–5.32)	0.021	2.43	(1.23–5.74)	0.024
>50 mm	4.38	(1.92–10)	<0.001	1.98	(1.08–2.87)	0.042
**Pathological differentiation**						
Well	1		Ref.			
Moderate	2.12	(0.85–5.31)	0.109	2.13	(0.83–5.46)	0.117
Poor	23.17	(9.74–55.11)	<0.001	7.00	(2.75–17.84)	<0.001
Undifferentiated	12.83	(5.17–31.82)	<0.001	5.31	(1.98–14.2)	<0.001
**T stage**						
T1	1		Ref.	1		Ref.
T2	1.925	(1.264–2.93)	0.002	1.12	(0.53–2.37)	0.762
T3	3.445	(1.753–6.77)	<0.001	0.97	(0.39–2.42)	0.94
T4	6.881	(4.28–11.06)	<0.001	1.08	(0.46–2.55)	0.853
Tx	4.832	(3.17–7.37)	<0.001	1.02	(0.43–2.43)	0.97
**N**						
N0	1		Ref.	1		Ref.
N1	9.83	(5.26–18.4)	<0.001	2.02	(0.98–4.16)	0.056
N2	14.4	(8.47–24.5)	<0.001	2.52	(1.3–4.89)	0.006
N3	21.07	(10.32–43.01)	<0.001	4.41	(1.9–10.23)	0.001
Nx	18.02	(10.46–31.04)	<0.001	3.37	(1.57–7.25)	0.002
**M**						
M0	1		Ref.	1		Ref.
M1	12.58	(8.42–18.81)	<0.001	2.17	(1.31–3.62)	0.003
Mx	6.15	(2.65–14.28)	<0.001	0.66	(0.23–1.85)	0.426
**Therapy**						
Surgery	1		Ref.	1		Ref.
Radiation/chemotherapy	11.95	(7.57–18.85)	<0.001	4.31	(2.25–8.28)	<0.001
Combined therapy	10.2	(7.3–15.74)	<0.001	2.63	(1.65–4.18)	<0.001
No defined therapy	9.69	(5.68–16.5)	<0.001	5.97	(2.95–12.08)	<0.001

### Nomogram Model and Validations

**Figure 4:**
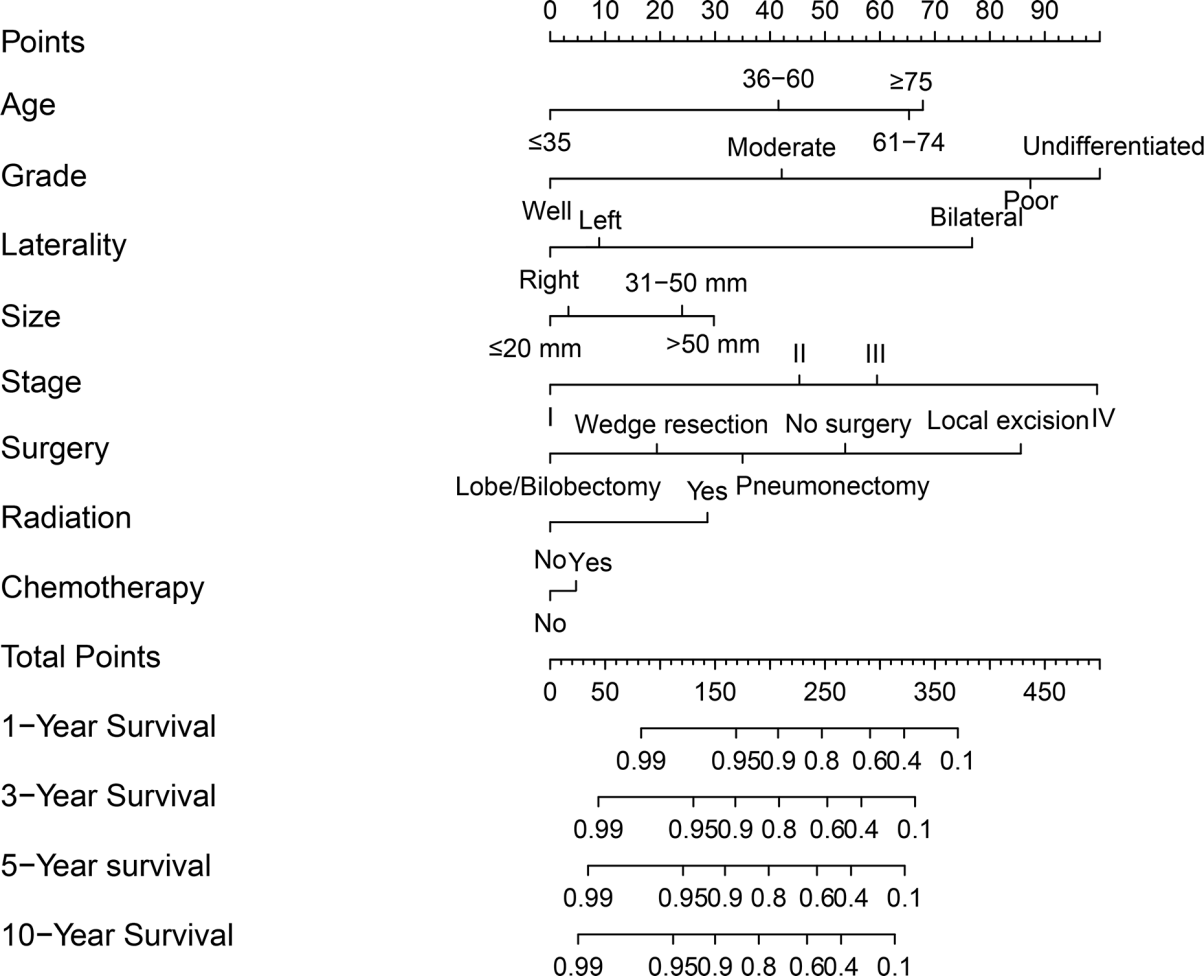
A nomogram of predicting the 1-year, 3-year, 5-year, and 10-year cancer-specific survival rates for PMEC patients.

**Figure 5:**
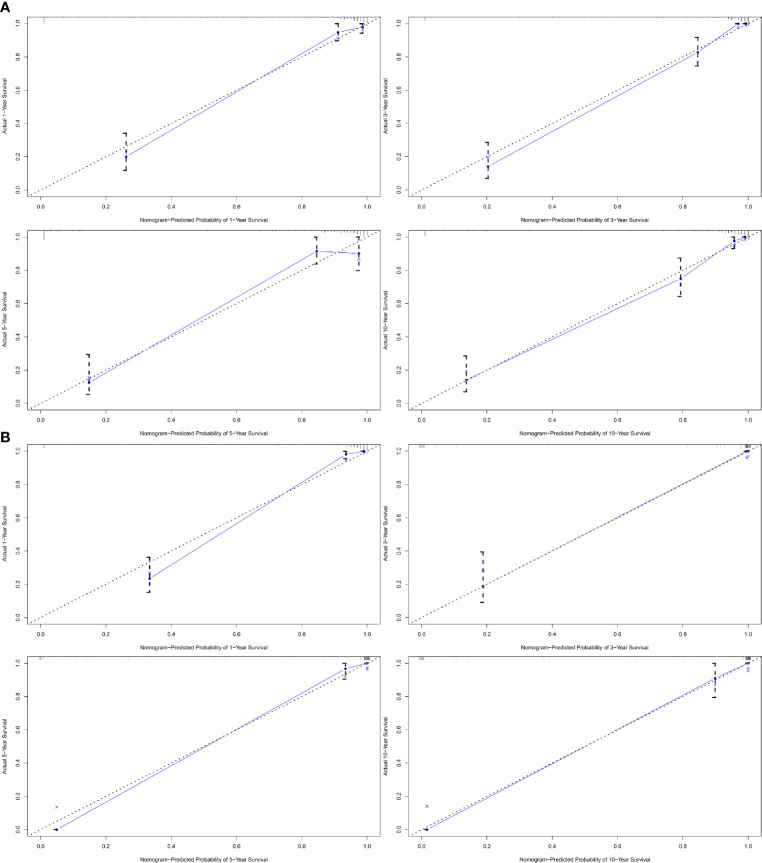
The calibration curve of nomogram-predicted probability. **(A1)** The calibration curve of 1-year cancer-specific survival outcomes. **(A2)** The calibration curve of 3-year cancer-specific survival outcomes. **(A3)** The calibration curve of 5-year cancer-specific survival outcomes. **(A4)** The calibration curve of 10-year cancer-specific survival outcomes. **(B1)** The validation curve of 1-year cancer-specific survival outcomes. **(B2)** The validation curve of 3-year cancer-specific survival outcomes. **(B3)** The validation curve of 5-year cancer-specific survival outcomes. **(B4)** The validation curve of 10-year cancer-specific survival outcomes.

A nomogram was established based on results of multivariate Cox analysis (**Figure 4**). The C-index of this nomogram was 0.921, indicating that the model was reliable (**Figure 4**). In addition, the calibration curve showed that the predicted curve and the actual observation curve were very close, which indicated that the result was reliable (**Figure 5**). The validation cohort was utilized to verify the nomogram and its C-index was 0.968 (**Figure 5**). The calibration curve showed high favorable consistency, indicating that the nomogram could be trusted.

## Discussion

PMECs are rare pulmonary malignancies, and most PMEC studies are small series or single case reports due to their scarcity. Therefore, characteristics and outcomes of this disease remain unclear. The SEER database is one of the largest cancer registries worldwide. It covers a wide range of data on patients, tumors, treatments, and survival outcomes. In the present study, we examined the characteristics and prognostic factors of PMECs and identified variables affecting survival, utilizing data from the SEER database between 1975 and 2016. We constructed a predictive nomogram of PMEC for survival rates in this study.

Consistent with previous reports, our study showed that there was a similar distribution of PMEC incidence between males and females (12, 14). It was previously observed that the onset age of MEC ranged from 4 years old to 86 years old, and nearly 50% of patients were below 40 years old (12, 14, 15). Similar to other types of MEC, PMEC still showed a similar trend of younger-onset (59.5% of patients were under 60, and 40.5% of patients were over 60) in our study. The vast majority of SGTs are endobronchial lesions, predominantly involving the stem bronchi, carina, and trachea (6, 12, 16). More than 66% of patients with SGTs have endobronchial masses (12). However, we observed that PMEC most frequently occurred in the upper lobes (35.2%) and lower lobes (33.1%), instead of in the bronchus. This result aligned with previous database-based PMEC studies (13) and revealed the diversity of its characteristics.

PMECs are a pathological subtype of p-SGTs, representing a rare category of lung carcinomas (13). Pulmonary salivary gland tumors (p-SGTs) were first identified as early as the 1950s and were initially classified as benign bronchial adenomas (17). In the 1960s, researchers observed that this broad group of tumors were factually capable of invasive growth and metastasis but were more indolent than the prevalent neoplasms of bronchial origin (18). The prognosis of MEC patients is generally considered optimistic. A previous large series suggested that the 5-year and 10-year OS rates of p-SGTs were 80.0 and 62.7%, respectively. Our results found that the 5-year CSS and OS rates of patients with PMEC stage I–II were 94.2 and 85.5%, respectively. The 5-year CSS and OS rates of patients with PMEC stage III–IV disease were 32.1 and 23.8%, respectively. The survival outcomes of patients with PMEC seemed to be better than those of patients with non-small cell lung cancer (NSCLC) and small-cell lung cancer (SCLC). It was reported that the 5-year survival rate of all NSCLC patients was 52.6% (19). The 5-year overall survival of NSCLC in different stages was as follows: IA, 66%; IB, 53%; IIA, 42%; IIB, 36%; IIIA, 10%; IIIB, 12%; and IV, 4% (20). For small cell lung cancer, it was reported that the 5-year survival rate of patients was 22% for local disease and 1% for extensive disease [13]. PMECs belong to low-grade lung malignancies and are considered to have a more favorable survival outcome than NSCLC and SCLC. However, the 3- and 5-CSS rates of patients with stage III–IV PMEC were only 39.45 and 32.1%, respectively, in our study. The results of this study revealed that the prognosis is not good for advanced patients with PMEC. Considering that our data span a large number of years, we analyzed data from the past ten years and ten years ago. A difference was observed in the survival curves of the two periods of time. The results may be related to living conditions, pharmaceutical treatments and medical conditions in the past decade.

The prognostic factors of MEC are controversial. Important prognostic factors of MEC include age, TNM stage and pathological differentiation grade (21–23). Poor OS was associated with older age, higher clinical stages, larger tumour size and non-surgical treatments (24–26). Our study found that the survival of PMEC patients was not only significantly affected by factors of age, pathological differentiation, TNM stage, tumor size, lymph node metastasis, distant metastasis and treatment approaches. The primary tumor site was also identified as a survival-associated factor. Moreover, age, bilateral tumors, tumor size, pathological differentiation grade, TNM stage, lymph node metastasis and treatment approaches were independent prognostic factors for PMEC in the current study. In addition, our study revealed that age >60, poor differentiation, tumor sizes >30 mm, lymph node metastases and distant metastases were especially associated with worse survival outcomes.

Previously, surgical resection was considered the most effective treatment for MEC (6, 26). However, the usefulness of chemotherapy and radiotherapy for advanced diseases remains controversial (23). In our study, considering the significant difference in survival between stage I–II and stage III–IV patients, we separated stage I–II and stage III–IV patients and examined the effect of treatment options on their survival curve alone. Our results revealed that cancer-directed surgical resection was the main treatment for stage I–II PMEC. It had excellent long-term survival outcomes in both CSS and OS. In patients with stage III–IV PMEC, surgical treatments (surgery alone and surgery plus radiation and/or chemotherapy) were still associated with improved survival outcomes compared with radiation and/or chemotherapy alone. Surgical resection treatment was the optimal treatment option for PMEC patients. This finding is in agreement with the previous research results (26–28). Furthermore, we further analyzed the impact of surgical options on survival. We found that patients who chose local surgical resection had the worst survival, while patients who chose pneumonectomy, lobectomy, or wedge procedures obtained good survival benefits. Therefore, we believe that for PMEC patients, such as MEC patients, surgery is still the best treatment option. However, local surgical resection should be avoided. Surgical treatment is associated with obviously improved survival outcomes. Pneumonectomy, lobectomy, and wedge procedures should be the top three recommended surgical options, but which surgical option should be chosen is based on the actual situation.

In the past, survival predictions for PMEC were based only on the predictive nomogram of SGTs or MEC (13, 25). No separate PMEC nomogram from a large series of model studies had been constructed. Since CSS may be more representative of the nature of the tumor itself than OS, we constructed a nomogram of PMEC CSS to forecast the 1-, 3-, 5- and 10-year CSS rates of PMEC patients. This is the first time that PMEC-specific nomogram was constructed. The C-index of this nomogram in the training and validation cohorts reached 0.921 and 0.968, respectively. The C-index and calibration curves showed the strength of this model. Validations of this model also supported the reliability and accuracy of the nomogram. Therefore, we believe that our model can provide clinicians with good model predictions for individual PMEC patients.

Even though a total of 585 clinical cases of PMEC were included and analyzed in our study, there were still some limitations. First, this study was retrospective, thus there were some inevitable confounding factors that affected the accuracy of our results. Second, our results were conducted with the data from the SEER database. These results might only represent American patients and might not be applicable to all PMEC patients worldwide. Third, with the limited data on PMEC patients, our study did not conduct validations for the main outcomes. Consequently, the reliability and accuracy of our results might have been affected and need to be reevaluated by future studies. Finally, adverse effects of treatments, quality of life, *etc.* were not analyzed in the current study. This may have caused our analysis to be incomplete. Therefore, high-quality studies are still needed in the future to evaluate clinical and survival outcomes of PMEC patients.

## Data Availability Statement

Publicly available datasets were analyzed in this study. This data can be found here: Surveillance, Epidemiology, and End Results (SEER) database (https://seer.cancer.gov/).

## Ethics Statement

Ethical review and approval were not required for the study on human participants in accordance with the local legislation and institutional requirements. Written informed consent for participation was not required for this study in accordance with the national legislation and the institutional requirements.

## Author Contributions

LQ, PS, and GZ conceived the idea and edited the manuscript. LQ, PS, PC, and MS wrote the manuscript. XS, CH, and NL contributed to literature search. LQ, PS, and G-cG were involved in data collection and classification. LQ, PS, FL, and HJ prepared the tables and figures. HW and GZ supervised the final version of the manuscript. All authors contributed to the article and approved the submitted version.

## Funding

This study was supported by grants from National Natural Science Foundation of China (Grant No. 81874042 and Grant No. 81972182).

## Conflict of Interest

The authors declare that the research was conducted in the absence of any commercial or financial relationships that could be construed as a potential conflict of interest.
