# Corrigendum: Clinical characteristics and prognosis of patients with pulmonary mucoepidermoid carcinoma: a SEER-based analysis

**DOI:** 10.3389/fonc.2024.1486678

**Published:** 2024-09-13

**Authors:** Lingxiao Qiu, Pan Song, Pingmei Chen, Huaqi Wang, Fangfang Li, Mengxuan Shu, Gen-cheng Gong, Xiangjin Song, Chun Huang, Hongxia Jia, Nana Li, Guojun Zhang

**Affiliations:** ^1^ Department of Respiratory Medicine, The First Affiliated Hospital of Zhengzhou University, Zhengzhou, China; ^2^ Academy of Medical Sciences, Zhengzhou University, Zhengzhou, China; ^3^ Department of Urology, West China Hospital of Sichuan University, Chengdu, China; ^4^ Department of Neonatology, West China Guang’an Hospital, Sichuan University, Guang’an, China; ^5^ Zhengzhou Key Laboratory for Chronic Respiratory Disease, Zhengzhou, China; ^6^ Henan Provincial Respiratory Medicine Center, Zhengzhou, China; ^7^ The First Clinical Medical College of Lanzhou University, Lanzhou, China; ^8^ State Key Laboratory of Respiratory Disease, National Clinical Research Center for Respiratory Disease, Guangzhou Institute of Respiratory Health, The First Affiliated Hospital of Guangzhou Medical University, Guangzhou, China

**Keywords:** pulmonary mucinous epidermoid carcinoma, clinical characteristics, prognosis, Surveillance, Epidemiology, and End Results (SEER), nomogram

In the published article, there was an error. In the “*Methods*” section of the **Abstract**, an error was present. It was my oversight that led to the incorrect date being written.

A correction has been made to **Abstract**, *Methods*, Paragraph 1. This sentence previously stated:

“In the Surveillance, Epidemiology, and End Results database from January 1, 2016 to December 31, 2016, patients pathologically diagnosed with PMEC were identified.”

The corrected sentence appears below:

“In the Surveillance, Epidemiology, and End Results database from January 1, 1975 to December 31, 2016, patients pathologically diagnosed with PMEC were identified.”

The authors apologize for this error and state that this does not change the scientific conclusions of the article in any way. The original article has been updated.

